# A Virginia Mountain City Responds to the Challenge of Improving Health Outcomes

**DOI:** 10.13023/jah.0103.04

**Published:** 2019-09-27

**Authors:** Robert S. Cowell

**Affiliations:** City of Roanoke, Virginia, bob.cowell@roanokeva.gov

**Keywords:** health outcomes, Virginia, social determinants, post-industrial city

## Abstract

In 2012, Roanoke Virginia was becoming a city of haves and have-nots, a place where many were benefitting from revitalization underway but too many were seeing their situation grow worse and becoming even more entrenched. Poverty with levels as high as 50% in some neighborhoods; life expectancy sometimes 14 years shorter than those living just one or two neighborhoods over; and lack of access to fresh food, medical care, and economic opportunities—all within view of the largest hospital in the region was unacceptable.

Upon arrival a couple years ago, I found a city celebrating its transformation from a post-industrial Appalachian town once dependent on now-shuttered manufacturing and railroad shops to a vibrant center of healthcare and bio-medical research, an outdoor recreation hub tucked in among the beauty of Virginia’s Blue Ridge. I found a small city taking on some daunting challenges. You see, while undergoing this transformation, vestiges of its history still gnawed away at progress. It’s long-lasting dependency on manufacturing and segregationist past had led to deep inequities in access to health care, life expectancy, and economic opportunity.

The 2012 Community Health Needs Assessment, prepared by the regional healthcare provider and partners, revealed through data and statistics an unavoidable and uncomfortable truth. Roanoke Virginia was becoming a city of haves and have-nots, a place where many were benefitting from revitalization underway but too many were seeing their situation grow worse and becoming even more entrenched. Poverty with levels as high as 50% in some neighborhoods; life expectancy sometimes 14 years shorter than those living just one or two neighborhoods over; and lack of access to fresh food, medical care, and economic opportunities—all within view of the largest hospital in the region was unacceptable.

Using urgency generated by the Needs Assessment, community leaders crafted a response. There needed to be a framework and a backbone organization, and the approach needed to be collaborative, place-based, data-driven, and community-led. The backbone organization came to be called Healthy Roanoke Valley, a new start-up bringing together more than 50 organizations, financially and administratively supported by the local United Way and Carilion Clinic. Healthy Roanoke Valley’s mission was to mobilize community resources to improve access to care, coordinate services, and promote a culture of wellness.

The framework selected would simultaneously address access to health care and social determinants of health outcomes. The Robert Wood Johnson Foundation framework for what influences health served in this capacity, emphasizing health behaviors, social and economic factors, clinical care access and quality, and the physical environment.

Based on the Needs Assessment and intentional conversations with those residing in the two federally designated medically underserved portions of the community, it was determined that the initial focus would (1) bring needed healthcare services directly to the people, (2) address the physical quality and health of neighborhoods, (3) increase access to and affordability of fresh and healthy food, and (4) increase educational opportunities with a specific focus on youth. Supported by the backbone of Healthy Roanoke Valley, local nonprofits, the city government, Carilion Clinic, and others went to work.

## HEALTHCARE SERVICES

The Needs Assessment and research had revealed that even though two major hospitals operated a very short distance from the most challenged neighborhoods, accessing medical services remained an issue. Transportation difficulties, lack of insurance, and other limitations necessitated a bold move. Led by Healthy Roanoke Valley a partnership including Carilion Clinic, New Horizons, the Bradley Free Clinic, and others opened new clinics and offered additional services. The G. Wayne Fralin Free Clinic opened in proximity to the community’s homeless shelter, offering free medical, dental, and mental health services. The New Horizons Health Center opened a 32,000 square-foot clinic in the historically African-American portion of the city, offering free or low-cost medical, dental, and mental health services and operating a pharmacy. In addition to financial support, Carilion Clinic staff conducted hundreds of health outreach events throughout the community assisting thousands with securing needed medical care.

## HEALTHY NEIGHBORHOODS

Early in the process it was evident that the health and vitality of neighborhoods was playing a major role in determining health outcomes for residents. Safe and affordable housing, access to recreation, and the quality of the physical environment all played a role. In response, the city began targeting the majority of its federal community development funds in a single area for an extended time. This focus enabled a large portion of the community’s annual allocation of federal funds (generally somewhere in the vicinity of $1 million) to be leveraged with funds from area nonprofits to have a greater impact. In the current target area this enabled the City of Roanoke, Habitat for Humanity in Roanoke Valley, and Total Action for Progress (a community action agency), and Renovation Alliance to renovate or construct over 100 homes and remove lead from another dozen. This targeting effort also enabled the transformation of what had been a crime-ridden nightclub into a Community Center providing a food bank, culinary training facility, and community meeting space, operated by Feeding America Southwest Virginia.

## ACCESS TO HEALTHY AND FRESH FOODS

With large swaths of the city located in food deserts with little access to quality fresh healthy food, Healthy Roanoke Valley in partnership with Carilion Clinic, Feeding America Southwest Virginia, Local Environmental Agriculture Project (LEAP), and the City of Roanoke embarked on several initiatives. An urban farm, community gardens, and farmers markets bring healthy fresh food in proximity to area residents. An incentive program pioneered by LEAP and financially supported by Carilion Clinic provide extra value for SNAP, Medicaid, and WIC recipients, helping ensure that fresh healthy food is not only available but also affordable. LEAP also deploys a mobile market making stops at local retirement centers, healthcare clinics, and other locations. Feeding America Southwest Virginia, YMCA of Virginia’s Blue Ridge, and the City of Roanoke collaborate to ensure that during the summer, every child in the community has access to healthy food by providing meals at neighborhood library branches as part of the Star City Feed and Read initiative, an effort recognized by the Campaign for Grade-Level Reading and the National Civic League as exemplary.

## ECONOMIC OPPORTUNITIES THROUGH EDUCATION

Poverty and the inability to break free of its grip continued to impede opportunity. Knowing that educational attainment plays a significant role in generational poverty, the City of Roanoke and the Roanoke City Public Schools collaborated with dozens of partners to develop the Star City Reads Campaign with the goal of improving proficiency in 3rd grade–level reading, an accomplishment demonstrated to increase academic success throughout a person’s life. Through the distribution of nearly 100,000 free books, various reading programs, the summer feed and read program, and others, proficiency levels have increased nearly 10% in just 5 years, an accomplishment earning the City of Roanoke its seventh All-America Award from the National Civic League and the honor of being the first city placed in its Hall of Fame. In addition to improving reading skills, the City in partnership with Goodwill of the Valleys conducts a summer youth employment program where middle and high school students receive job training and paid employment opportunities through the summer. This program provides participants with much-needed financial resources as well as skills that will benefit them after graduation.

## PROGRESSING BUT SLOWLY

Through a collective approach these initiatives have yielded progress. The most recent Community Health Needs Assessment (2018) noted improvements in access to medical care and healthy food, utilization of health screenings and preventive practices, and reduced emergency room visits. Further, the community saw improvement in its Robert Wood Johnson Health Factor Rankings for the 6th consecutive year. Still, progress has been frustratingly slow with key variables—poverty for example, seemingly not improved at all. As a result, most recently there has been a “doubling down” of efforts. The local United Way has completely altered its service delivery model focusing on building partnerships that collaboratively address issues rather than just funding projects and established the audacious goal of lifting 10,000 families out of poverty. The City has placed equity and health outcomes at the core of its Comprehensive Plan update and launched a Financial Empowerment Center to help residents enhance their financial well-being. Carilion has begun development of a new wellness clinic embedded in a local elementary school, piloting what they hope to be a model of service delivery throughout the region. Perhaps most aggressive of all is an effort stimulated by the National League of Cities through their Cities of Opportunity initiative where the City of Roanoke, Carilion Clinic and Virginia Tech-Carilion seek to strengthen the link between the success of the City’s residents and families to that of the growth of the community’s anchor institutions.

This is where I find myself 2 years in: a city at the point of accelerated and deeper emphasis, building on the success of the past few years and current momentum hoping to transform the lives of every person residing in this community. There have been lessons learned along the way. A catalyzing set of data and compelling narrative are essential. A clear set of objectives guided by evidence-based solutions within a recognized framework led by a backbone entity collaborating with many is critical. Perhaps most notable is the need to be bold—lives are at stake as is the future of many of our communities. Now is not the time to be timid or to maintain the status quo. Too much and too many are depending on us. Our efforts should enable us to rise above our history, to help us create healthy and happy lives full of opportunity so that we may be worthy of the beauty that surrounds so many of our communities.

